# Microvascular complications burden (nephropathy, retinopathy and peripheral polyneuropathy) affects risk of major vascular events and all-cause mortality in type 1 diabetes: a 10-year follow-up study

**DOI:** 10.1186/s12933-019-0961-7

**Published:** 2019-11-16

**Authors:** Monia Garofolo, Elisa Gualdani, Rosa Giannarelli, Michele Aragona, Fabrizio Campi, Daniela Lucchesi, Giuseppe Daniele, Roberto Miccoli, Paolo Francesconi, Stefano Del Prato, Giuseppe Penno

**Affiliations:** 1 Section of Diabetes and Metabolic Disease, Department of Clinical and Experimental Medicine, University of Pisa and Azienda Ospedaliero-Universitaria Pisana, Via Paradisa, 2, 56124 Pisa, Italy; 2 Epidemiology Unit, Regional Health Agency (ARS) of Tuscany, Florence, Italy

**Keywords:** Type 1 diabetes mellitus, Microvascular complications, Microvascular burden, Diabetic kidney disease, Diabetic retinopathy, Peripheral diabetic polyneuropathy, All-cause mortality, Cardiovascular disease

## Abstract

**Background:**

Microvascular complications (MC) have been claimed to increase the risk for cardiovascular disease in diabetic subjects. However, the effect of MC burden on the risk of major vascular outcomes and all-cause mortality in type 1 diabetes is still poorly explored. We evaluated the relationship between microvascular complications burden and incidence of major cardiovascular events and all-cause mortality in subjects with type 1 diabetes.

**Methods:**

We recruited 774 participants with type 1 diabetes in a single-center observational study over a follow-up of 10.8 ± 2.5 years. Hazard ratios (HR) for cardiovascular outcomes and all-cause death associated with microvascular complications were determined by unadjusted and adjusted Cox regression analysis.

**Results:**

Out of 774 individuals, 54.9% had no-MC, 32.3% 1 MC, 9.7% 2 MC and 3.1% 3 MC. A total of 54 deaths (7.0%) occurred. Death rate increased from no-MC 2.1% (Ref) to 1 MC 7.2% (HR 3.54 [95% CI 1.59–7.87]), 2 MC 14.7% (HR 6.41 [95% CI 2.65–15.49]) and 3 MC 66.7% (HR 41.73 [95% CI 18.42–94.57], p < 0.0001). After adjustments, HRs were: 1 MC 2.05 (95% CI 0.88–4.76), 2 MC 1.98 (95% CI 0.75–5.21), 3 MC 7.02 (95% CI 2.44–20.20, p = 0.002). Forty-nine subjects (6.7%) had at least one cardiovascular event, and cumulative incidence went from no-MC 2.2% (Ref) to 1 MC 5.0%; (HR 2.27 [95% CI 0.96–5.38]), 2 MC 26.8% (HR 12.88 [95% CI 5.82–28.50]) and 3 MC 40.9% (HR 29.34 [95% CI 11.59–74.25], p < 0.0001). Upon adjustments, HRs were: 1 MC 1.59 (95% CI 0.65–3.88), 2 MC 4.33 (95% CI 1.75–10.74), 3 MC 9.31 (95% CI 3.18–27.25, p < 0.0001). Thirty-five individuals (4.8%) had at least one coronary event, which cumulative incidence increased with MC burden (p < 0.0001).

**Conclusions:**

In type 1 diabetes, microvascular complications burden increases in an independent dose-dependent manner the risk of major cardiovascular outcomes and all-cause mortality. The presence and number of microvascular complications should be considered in stratifying overall cardiovascular risk in type 1 diabetes.

## Background

In spite of recent reports suggesting reduction of mortality rate among subjects with type 1 diabetes [1], mortality still remains higher compared to the general population [1] as recently reported in North America [2, 3], Europe [4–6] and Australia [7]. Petrie et al. reported that Swedish individuals with type 1 diabetes have a life expectancy (LE) gap at age 20 of about 10–12 years compared with the general population [6]. Consistently, Huo et al. show that, at birth, Australian subjects with type 1 diabetes have an estimated LE loss of 12.2 years compared with those without diabetes [7].

This excess risk persists even if all cardiovascular (CV) risk factors are properly controlled [8]. In particular it has been calculated that the risk of all-cause as well as cardiovascular death in type 1 diabetic subjects with good glycaemic control remains twice as much the risks in the general population [9].

These findings highlight the need to improve risk stratification among type 1 diabetic subjects in the attempt to identify those with very high risk. To this purpose, risk prediction models based on easily accessible patient’s clinical characteristics have been proposed [10, 11].

Epidemiological studies suggest that the presence of microvascular complications can increase the risk of CV disease and overall mortality in type 1 diabetes [12, 13]. In the Joslin 50-year Medalist Study and in the Finnish Diabetic Nephropathy Study (FinnDiane) the coexistence of proliferative diabetic retinopathy (PDR) and stage ≥ 3b diabetic kidney disease (DKD) was associated with higher prevalence of CV disease in patients with long-standing type 1 diabetes [14]. Nonetheless, till recently [15], cross-sectional [14, 16] and prospective data [17] assessing the impact of cumulative microvascular disease burden on CV disease and overall mortality in type 1 diabetes have been scanty.

Therefore, we have investigated to which extent the cumulative burden of retinopathy, nephropathy, and peripheral neuropathy is associated with the incidence of all-cause mortality, major CV disease and coronary heart disease (CHD) irrespective of conventional CV risk factors in individuals with type 1 diabetes. Our hypothesis is that the increasing microvascular complication burden, i.e. the number of microvascular complications, can increase the risk of all-cause mortality and incidence of CV events, being the highest in those with complications in all three microvascular beds.

## Methods

### Study design and population

This is an observational, cross-sectional, single-centre study with baseline CV risk stratification and prospective assessment of all-cause mortality and major vascular outcomes in a cohort of individuals with type 1 diabetes. The research design and study population have been previously described [18]. All subjects (n = 843) attending the Diabetes Outpatient Clinic of the Azienda Ospedaliero-Universitaria Pisana from January 1, 2001 to December 31, 2009 for their usual screening for diabetic complications were considered eligible. Type 1 diabetes was diagnosed on the basis of age at diagnosis < 36 years, immediate requirement of insulin therapy and unbroken need for insulin therapy for the first year after diagnosis [19]. Pregnant women, participants of not-white ethnicity, those with diabetes duration < 1 year, those on dialysis or with renal transplantation, and three individuals for whom valid information on vital status could not be retrieved were excluded. Finally, a total of 774 individuals (52.6% men; age 40.2 ± 11.7 years; duration of diabetes 19.4 ± 12.2 years, mean ± SD) entered the study. The Ethics Committee of the University of Pisa approved the study protocol, consent procedures, and data analysis plan.

Information about onset and duration of diabetes (DD), smoking habits, insulin therapy, and blood pressure- and lipid-lowering therapies were collected for all subjects along with body mass index (BMI), waist-to-hip ratio (WHR), and resting blood pressure (BP). Hypertension was defined as systolic BP > 140 mmHg and/or diastolic BP > 80 mmHg and/or use of antihypertensive medications.

Serum creatinine, glycated haemoglobin (HbA_1c_), total- and HDL-cholesterol, triacylglycerol, alanine aminotransferase (ALT), aspartate aminotransferase (AST), gamma-glutamyltransferase (GGT), uric acid and fibrinogen were determined in all subjects at the time of first screening for diabetic complications.

### Baseline measurements

Albumin-to-creatinine ratio (ACR) was determined in at least three first-voided urine samples obtained with at least 1-month interval in the year preceding the recruitment. Urine samples (n = 175, 6.7%) with abnormal sediment (nitrites or ≥ 250 leucocytes/ml) were discarded. All other samples were assayed for albumin (BNII; Dade Behring Diagnostic, Marburg, Germany; intra- and inter-assay variation < 2.0% and < 3.5%, respectively) and creatinine (modified Jaffé reaction) on the same morning of collection.

HbA_1c_ was measured by high-performance liquid chromatography using DCCT-aligned methods [20]; triacylglycerol, total- and HDL-cholesterol were determined by colorimetric enzymatic methods, and LDL-cholesterol was calculated by the Friedewald formula [21]. Standard clinical laboratory methods have been employed for the measurement of serum creatinine, ALT, AST, GGT, uric acid and fibrinogen.

### Assessment of diabetes complications

Diabetic kidney disease was established based on the geometric mean of three ACR values, and estimated glomerular filtration rate (eGFR) was calculated by the Chronic Kidney Disease Epidemiology Collaboration (CKD-EPI) equation [22]. DKD was diagnosed on the basis of ACR > 3.4 mg/mmol [23] and/or eGFR < 60 mL/min/1.73 m^2^ [24].

The presence and severity of diabetic retinopathy (DR) was assessed by retinal photography using a wide-angle (45°) midriatic camera. Photos of disc-macula-temporal and disc-nasal regions were taken [25] and the eye with worse retinal condition including previous photocoagulation or surgical treatment, was used for retinopathy staging: absent, mild, moderate or severe non-proliferative, proliferative diabetic retinopathy, or maculopathy, according to the Global Diabetic Retinopathy Project Group criteria [26]. For further analysis, individuals with non-proliferative retinopathy of mild or moderate degree were classified as non-advanced diabetic retinopathy, whereas those with severe non-proliferative, proliferative, maculopathy, or blindness were grouped into the advanced, sight-threatening diabetic retinopathy category.

Diabetic peripheral neuropathy was defined on the presence of symptoms and signs assessed by the Michigan Neuropathy Screening Instrument questionnaire designed to identify symptoms of muscle weakness and sensory dysfunction [19, 27], by the examination of the feet for neuropathic ulcerations, by reduced or absent knee and ankle reflexes, and/or by measurement of vibration perception threshold using a biothesiometer applied bilaterally at the medial malleolus and the tip of the big toe [19, 27]. The mean of three readings at 10 s intervals on each site was calculated and interpreted according to age-specific reference values [28].

Presence of previous CV disease was ascertained on the basis of medical history of any major acute CV events, i.e. myocardial infarction, stroke, ischemic foot ulcer or gangrene, amputation and coronary, carotid, and/or lower limb revascularization. A 12 lead resting ECG was recorded and coded according to the Minnesota Code [29]. Peripheral vascular disease was defined on a positive history of ischemic ulceration, gangrene, amputation or lower limb revascularization, or diagnosed on the presence of reduced or absent femoral and/or foot pulses and reduced ankle/brachial pressure ratio (< 0.9).

### Calculation of the EURODIAB PCS risk score

The EURODIAB Prospective Complications Study (PCS) risk score was calculated as proposed by Soedamah-Muthu et al. [10]. The score calculates risk for major outcomes (CHD, stroke, end stage renal disease, amputation, blindness) and all-cause death in type 1 diabetes on the basis of age, HbA_1c_, WHR, ACR, and HDL levels allowing risk stratification as low (LS, ≤ 15), intermediate (IS, 16 to 19) and high score (HS, ≥ 20).

### Assessment of outcomes

The primary outcome was the time to all-cause mortality. Follow-up data for each patients were verified up to December 31, 2017. Vital status was available for all participants and was verified over a mean follow-up of 10.8 ± 2.5 years (median 10.2, IQR 9.2–12.3) by interrogation of the Italian Health Card Database (http://sistemats1.sanita.finanze.it/wps/portal/). The secondary outcomes were the time to first major CV event (i.e. occurrence of first event of myocardial infarction, coronary revascularization, stroke, carotid revascularization, and ulcer, gangrene, amputation and peripheral revascularization) and the time to first coronary event (i.e. occurrence of first event of myocardial infarction or coronary revascularization). Data for major CV and coronary events were available for 736 participants (95.1%), and were obtained, upon data anonymization (in compliance with current privacy legislation), in collaboration with ARS Toscana (Regional Health Agency of the Tuscany Region) through hospital discharge registers. ICD-9-CM codes (International Classification of Diseases, 9th Edition, Clinical Modification) was used to detect major CV and coronary artery events (Additional file 1: Table S1). All events (for primary and secondary outcomes) occurring between date of enrollment and end of follow-up or death were considered as incident.

### Statistical analyses

Data are expressed as median (interquartile range, IQR) and/or mean ± SD for continuous variables, and number of cases and percentage for categorical variables. Continuous variables were compared by the Student’s t-test or one-way ANOVA (Welch robust test for equality of means when appropriate based on the Levene statistic) for normally distributed variables. Wilcoxon Sum-of-Ranks (Mann–Whitney) U test or Kruskal–Wallis tests were used for variables with skewed distribution. Pearson χ^2^ or the Fisher exact tests were applied to categories. The Spearman’s rank-order correlation was run to measure strength and direction of associations between two variables measured on ordinal scale. For post hoc comparisons, Scheffe’s test or Tamhane’s test, Mann–Whitney U test and χ^2^ test were used for normally distributed, not-normally distributed, and categorical variables, respectively.

Crude mortality rates and incidence of outcomes were described as events per 1000 person-years (PYs), with 95% exact Poisson Confidence Intervals (CI). Time to all-cause death or to each first outcome was plotted according to the MC groups as Kaplan–Meier (K–M) curves, and comparison was made using the log rank test. Univariate and multivariate Cox proportional hazard models were used to identify the effect of key covariates such as sex and age (model 1), or sex, age and cardiovascular risk factors (model 2), and sex, cardiovascular risk factors and the EURODIAB PCS risk score (model 3). Results are expressed as Hazard Ratio (HR) and 95% CI. Analyses have been performed in the whole cohort as well as in subjects free of CV disease at baseline (CVD−). A two-sided p value ≤ 0.05 was considered statistically significant.

A post hoc power calculation has been performed to evaluate the statistical power of our sample size for the primary outcome, i.e. all-cause mortality. Compared to the reference group (subject without MC), our groups of subjects with 1, 2 and 3 MC have a post hoc power of 87.5%, 97% and 100%, respectively, to detect the observed differences in mortality rate with an alpha error level of 5%.

All statistical analyses were performed using SPSS package 25.0 version (IBM SPSS, Chicago, IL).

## Results

We recruited 774 individuals with type 1 diabetes who have been followed up to a mean of 10.8 ± 2.5 years equivalent to 8387 PYs follow-up. At screening, 425 participants (54.9%) had no-MC, 250 (32.3%) had 1 MC, 75 (9.7%) 2 MC, and 24 (3.1%) 3 MC (Additional file 1: Figure S1). Distribution was unchanged (n = 418, 57.0%; n = 236, 32.2%; n = 62, 8.5% and n = 17, 2.3%, respectively; p = 0.620) upon exclusion of 41 subjects (5.3%) with prior CV events (CVD+).

Table 1 and Additional file 1: Table S2 show the baseline characteristics of the whole cohort. Overall the presence and severity of MC were associated with age, diabetes duration, BMI and WHR, HbA_1c_, systolic and diastolic BP, prevalence of hypertension, and rates of treatment with BP-lowering agents and renin-angiotensin system (RAS) blockers. On the contrary, there was no difference in gender distribution, smoking history, HDL-cholesterol, and triacylglycerol. Total- and LDL-cholesterol tended to be lower in individuals with 3 MC, most likely because of more common use of lipid-lowering therapy. MC burden was also associated with increasing levels of non-conventional risk factors such as uric acid and fibrinogen, lower eGFR and higher ACR (p < 0.0001 for all). Finally, the rate of previous CV events and prevalence of high-risk EURODIAB PCS score (≥ 20) also increased with MC (p < 0.0001 for both).

**Table 1:** Baseline characteristics of the study cohort, both overall and according to microvascular complications burden

	All patients (n = 774)	No MC (n = 425)	1 MC (n = 250)	2 MC (n = 75)	3 MC (n = 24)	p value
Men/women, n (%)	407/367 (52.6/47.4)	224/201 (52.7/47.3)	130/120 (52.0/48.0)	36/39 (48.0/52.0)	17/7 (70.8/29.2)	0.275
Age, years	40.2 ± 11.7	36.3 ± 10.1	43.0 ± 10.4***	48.4 ± 13.0*** ^††^	55.7 ± 13.7*** ^††† ‡^	< 0.0001
Age at diabetes diagnosis, years	20.8 ± 10.9	23.2 ± 10.7	17.5 ± 10.0***	17.7 ± 11.6***	24.6 ± 8.9^† ‡^	< 0.0001
Duration of diabetes, years	19.4 ± 12.2	13.1 ± 9.4	25.5 ± 10.2***	30.7 ± 11.2*** ^†††^	31.1 ± 12.9***	< 0.0001
BMI, kg/m^2^	24.8 ± 3.6	24.2 ± 3.3	25.3 ± 3.7***	26.2 ± 3.7***	26.8 ± 3.9**	< 0.0001
WHR	0.921 ± 0.063	0.914 ± 0.056	0.924 ± 0.069	0.943 ± 0.070**	0.945 ± 0.081	< 0.0001
Smoking habits (non-smokers, current smokers) (n. 759), n (%)	534/225 (70.4/29.6)	295/121 (70.9/29.1)	168/78 (68.3/31.7)	53/21 (71.6/28.4)	18/5 (78.3/21.7)	0.727
Fasting glucose, mmol/L	9.44 ± 4.56	9.10 ± 4.23	9.44 ± 4.78	10.74 ± 5.18*	11.44 ± 4.80	0.005
HbA_1c_, % (mmol/mol)	7.83 ± 1.18 (62.1 ± 12.9)	7.70 ± 1.19 (60.6 ± 13.0)	7.85 ± 1.11 (62.3 ± 12.2)	8.25 ± 1.10** (66.7 ± 12.0)	8.60 ± 1.30** ^†^ (70.4 ± 14.2)	< 0.0001
Systolic BP, mmHg	127 ± 18	122 ± 15	130 ± 18***	136 ± 20*** ^†^	150 ± 16*** ^††† ‡‡^	< 0.0001
Diastolic BP, mmHg	73 ± 9	72 ± 8	75 ± 9**	74 ± 10	79 ± 11**	< 0.0001
Total cholesterol, mmol/L	4.84 ± 0.88	4.72 ± 0.85	5.00 ± 0.90**	5.08 ± 0.85*	4.68 ± 1.07	< 0.0001
LDL cholesterol, mmol/L	3.01 ± 0.76	2.94 ± 0.76	3.11 ± 0.74*	3.12 ± 0.73	2.92 ± 0.94	0.019
HDL cholesterol, mmol/L						
Men	1.45 (1.24–1.71)	1.44 (1.22–1.71)	1.53 (1.29–1.71)	1.49 (1.32–1.68)	1.40 (1.06–1.73)	0.644
Women	1.74 (1.48–2.07)	1.71 (1.42–1.99)	1.79 (1.51–2.15)	1.79 (1.58–2.15)	1.76 (1.37–1.84)	0.246
Triacylglycerol, mmol/L						
Men	0.90 (0.71–1.24)	0.86 (0.69–1.15)	0.94 (0.71–1.30)	1.00 (0.81–1.68)	1.22 (0.81–1.62)	0.031
Women	0.78 (0.60–1.06)	0.75 (0.58–1.04)	0.80 (0.63–1.06)	0.84 (0.66–1.06)	1.32 (0.80–1.33)	0.706
ALT, U/L	20.0 ± 10.8	19.7 ± 11.5	20.1 ± 9.0	20.6 ± 8.9	23.6 ± 18.7	0.343
AST, U/L	22.3 ± 31.8	20.9 ± 16.1	24.4 ± 27.6	21.6 ± 10.9	26.7 ± 38.2	0.495
Gamma-GT, U/L	20.6 ± 33.3	18.4 ± 27.9	20.2 ± 20.5	22.5 ± 18.0	56.2 ± 125.8*** ^††† ‡‡‡^	< 0.0001
Uric acid, µmol/L	223.6 ± 67.4	213.7 ± 58.3	241.4 ± 72.3	316.3 ± 102.0*** ^†††^	381.7 ± 152.1*** ^††† ‡^	< 0.0001
Fibrinogen, µmol/L	9.89 ± 2.00	9.63 ± 1.93	10.93 ± 2.33	10.77 ± 1.59*** ^††^	12.32 ± 2.56*** ^††^	< 0.0001
Creatinine, µmol/L	73.3 ± 18.6	70.8 ± 12.8	72.0 ± 12.6	79.6 ± 24.9*** ^†^	111.3 ± 55.5*** ^††† ‡‡‡^	< 0.0001
Albumin-to-creatinine ratio (ACR), mg/mmol	0.49 (0.26–1.00)	0.40 (0.23–0.75)	0.52 (0.26–0.97)	1.66*** ^†††^ (0.40–8.38)	8.31*** ^††† ‡‡‡^ (3.67–51.08)	< 0.0001
eGFR, CKD-EPI, mL/min/1.73 m^2^	102.5 ± 17.4	107.4 ± 14.1	101.1 ± 13.8***	90.4 ± 21.9*** ^†††^	68.9 ± 28.0*** ^††† ‡‡‡^	< 0.0001
Daily insulin dose, IU/kg body weight	0.66 ± 0.20	0.66 ± 0.21	0.67 ± 0.19	0.66 ± 0.22	0.78 ± 0.21	0.070
MDI/CSII, n (%)	691/83 (89.3/10.7)	380/45 (89.4/10.6)	223/27 (89.2/10.8)	65/10 (86.7/13.3)	23/1 (95.8/4.2)	0.655
Treatment with BP-lowering agents, n (%)	151 (19.5)	28 (6.6)	61 (24.4)***	46 (61.3)*** ^†††^	16 (66.7)*** ^†††^	< 0.0001
Treatment with RAS blockers, n (%)	136 (17.6)	23 (5.4)	56 (22.4)***	42 (56.0)*** ^†††^	15 (62.5)*** ^†††^	< 0.0001
Treatment with lipid-lowering agents, n (%)	100 (12.9)	35 (8.2)	38 (15.2)**	18 (24.0)***	9 (37.5)*** ^††^	< 0.0001
Treatment with antiplatelet drugs, n (%)	50 (6.5)	6 (1.4)	19 (7.6)***	17 (22.7)*** ^†††^	8 (33.3)*** ^†††^	< 0.0001
Treatment with metformin, n (%)	46 (5.9)	18 (4.2)	20 (8.0)	5 (6.7)	3 (12.5)	0.110
Hypertension, n (%)	270 (34.9)	82 (19.3)	110 (44.0)***	55 (73.3)*** ^†††^	23 (95.8)*** ^††† ‡^	< 0.0001
Retinopathy: no retinopathy/non advanced/advanced, n (%)	452/202/120 (58.4/26.1/15.5)	425/0/0 (100/0/0)	26/164/60*** (10.4/65.6/24.0)	1/32/42*** ^†††^ (1.3/42.7/56.0)	0/6/18*** ^†††^ (0/25.0/75.0)	< 0.0001
Peripheral polyneuropathy: no/yes, n (%)	706/68 (91.2/8.8)	425/0 (100/0)	241/9*** (96.4/3.6)	40/35*** ^†††^ (53.3/46.7)	0/24*** ^††† ‡‡‡^ (0/100)	< 0.0001
Diabetic kidney disease: no/yes, n (%)	792/82 (89.4/10.6)	425/0 (100/0)	233/17*** (93.2/6.8)	34/41*** ^†††^ (45.3/54.7)	0/24*** ^††† ‡‡‡^ (0/100)	< 0.0001
Major adverse cardiovascular events, n (%)	41 (5.3)	7 (1.6)	14 (5.6)**	13 (17.3)*** ^††^	7 (29.2)*** ^†††^	< 0.0001
Coronary artery disease, n (%)	29 (3.7)	3 (0.7)	10 (4.0)**	10 (13.3)*** ^††^	6 (25.0)*** ^††^	< 0.0001
Stroke, n (%)	4 (0.5)	2 (0.5)	0 (0)	2 (2.7)	0 (0)	0.090
EURODIAB PCS risk score: LS, IS, HS, n (%)	466/205/103 (60.2/26.5/13.3)	324/84/17 (76.2/19.8/4.0)	127/87/36*** (50.8/34.8/14.4)	14/30/31*** ^†††^ (18.7/40.0/41.3)	1/4/19*** ^††† ‡‡^ (4.2/16.7/79.2)	< 0.0001
Cancer, n (%)	9 (1.2)	3 (0.7)	2 (0.8)	3 (4.0)* ^†^	1 (4.2)	0.042
Autoimmune thyreopathy, n (%)	113 (14.6)	58 (13.6)	44 (17.6)	8 (10.7)	3 (12.5)	0.372

Quantitative variables are shown as mean ± SD or median (IQR)

*MDI/CSII* multiple daily insulin injections/continuous subcutaneous insulin infusion

* p < 0.05; ** p < 0.01; *** p < 0.001 vs no MC

^†^p < 0.05; ^††^ p < 0.01; ^†††^ p < 0.001 vs 1 MC

^‡^p < 0.05; ^‡‡^ p < 0.01; ^‡‡‡^ p < 0.001 vs 2 MC

### Microvascular complications and all-cause mortality in the whole cohort

**Fig. 1:**
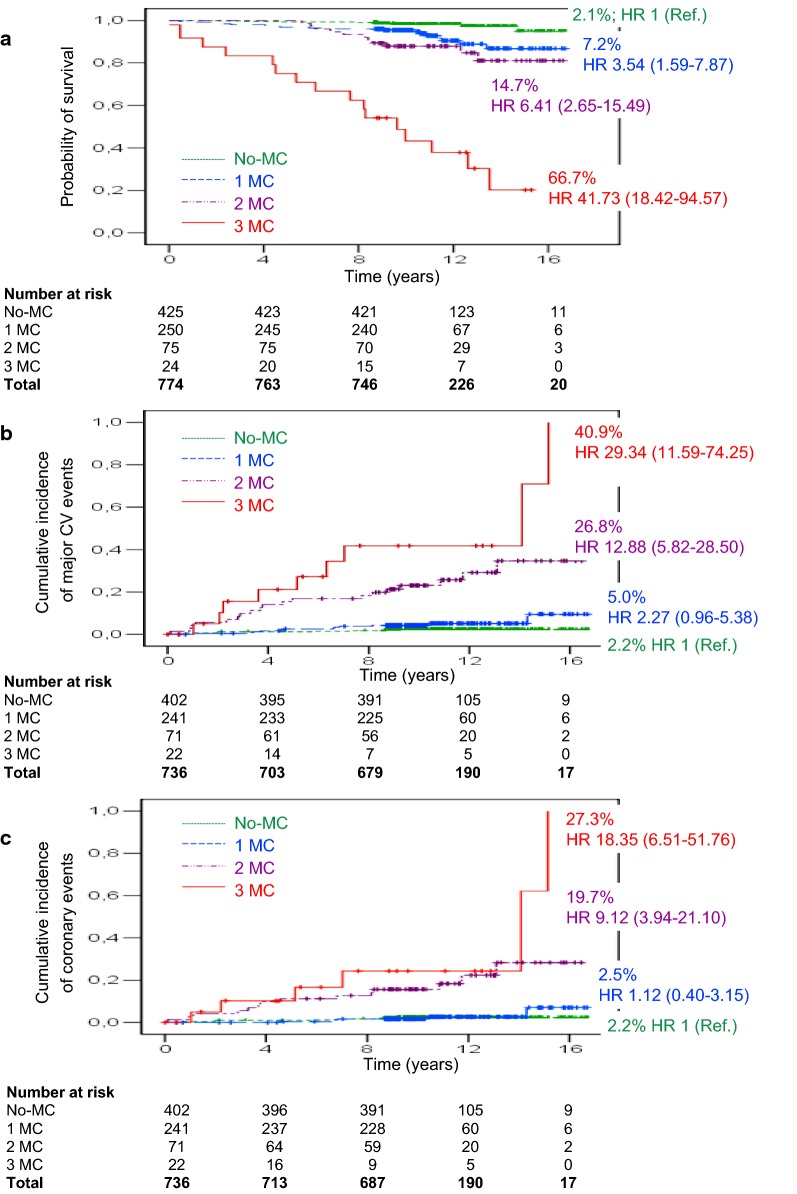
Kaplan–Meier curves for all-cause death and cumulative incidences of major vascular and coronary events by MC. no-MC: dotted green line, 1 MC: dashed blue line, 2 MC: dashdotted violet line, 3 MC: solid red line. Percentages of events and Cox proportional unadjusted HRs (95% CI) are shown for each MC group. **a** Survival from all-cause death: K–M Logrank 187.35, p < 0.0001. Pairwise over strata logrank statistic provides the following results: 1 MC vs. no-MC, logrank 10.96 (p = 0.0009); 2 MC vs. no-MC, logrank 22.77 (p < 0.0001); furthermore, 3 MC vs. no-MC, logrank 223.18, vs. 1 MC, logrank 78.94 and vs. 2 MC, logrank 30.62 (p < 0.0001, for all). **b** Cumulative incidence of major vascular events: K–M Logrank 127.61, p < 0.0001. Pairwise over strata logrank statistic provides the following results: 1 MC vs. no-MC, logrank 3.71 (p = 0.054); 2 MC vs. no-MC, logrank 67.22 and vs. 1 MC, logrank 28.49 (p < 0.0001, for both); furthermore, 3 MC vs. no-MC, logrank 110.38, vs. 1 MC, logrank 57.81 (p < 0.0001, for both) and vs. 2 MC, logrank 4.15 (p = 0.042). **c** Cumulative incidence of coronary events: K–M Logrank 81.47, p < 0.0001. Pairwise over strata logrank statistic provides the following results: 2 MC vs. no-MC, logrank 40.60 and vs. 1 MC, logrank 26.17 (p < 0.0001, for both); 3 MC vs. no-MC, logrank 55.23, vs. 1 MC, logrank 44.18 (p < 0.0001, for both)

**Fig. 2:**
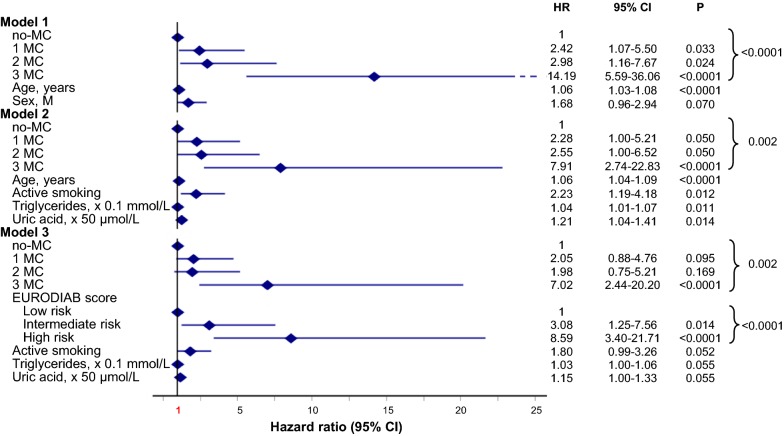
Adjusted HR for all-cause mortality by MC in the whole cohort. Model 1: adjusted for age and sex. Model 2: adjusted for age, sex, smoking habits, age at diagnosis of diabetes ≤ 18 years (n = 342; 44.2%), diabetes duration, BMI, HbA1c, LDL-cholesterol, HDL-cholesterol, triglycerides, uric acid, fibrinogen, hypertension and prior CV events. Model 3: adjusted for sex, smoking habits, age at diagnosis of diabetes ≤ 18 years, diabetes duration, LDL-cholesterol, triglycerides, uric acid, fibrinogen, hypertension, previous CV events and EURODIAB PCS risk score categories

During study follow-up 54 individuals (7.0%; 6.44 × 1000 PYs) died. Mortality increased with MC (p < 0.0001) going from 2.1% in subjects with no-MC (n = 9; 1.92 × 1000 PYs; Ref), to 7.2% in those with 1 MC (n = 18; 6.76 × 1000 PYs; HR 3.54 [95% CI 1.59–7.87]), 14.7% in those with 2 MC (n = 11; 12.93 × 1000 PYs; HR 6.41 [95% CI 2.65–15.49]), and 66.7% in individuals with 3 MC (n = 16; 79.77 × 1000 PYs; HR 41.73 [95% CI 18.42–94.57]; Fig. 1a). After adjustment for confounders (Fig. 2, Models 1 to 3), the HRs for primary outcome remained significant with the risk being the highest in those with 3 MC. In particular, in Model 3, HRs for death went from 2.05 in 1 MC (95% CI 0.88–4.76), to 1.98 (95% CI 0.75–5.21) in 2 MC, and 7.02 (95% CI 2.44–20.20, p = 0.002) in 3 MC, with independent effects for active smoking, triacylglycerol, uric acid and intermediate- (HR 3.08 [95% CI 1.25–7.56]) and high-risk EURODIAB PCS score (HR 8.59 [95% CI 3.40–21.71], p < 0.0001).

### Microvascular complications and all-cause mortality in subjects free of CV disease at baseline (CVD−)

**Fig. 3:**
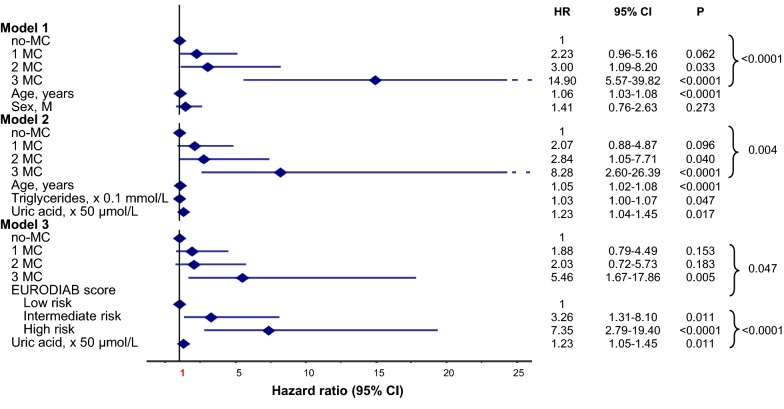
Adjusted HR for all-cause mortality by MC in CVD−. Model 1: adjusted for age and sex. Model 2: adjusted for age, sex, smoking habits, age at diagnosis of diabetes ≤ 18 years (n = 324; 44.2%), diabetes duration, BMI, HbA1c, LDL-cholesterol, HDL-cholesterol, triglycerides, uric acid, fibrinogen, hypertension and prior CV events. Model 3: adjusted for sex, smoking habits, age at diagnosis of diabetes ≤ 18 years, diabetes duration, LDL-cholesterol, triglycerides, uric acid, fibrinogen, hypertension, previous CV events and EURODIAB PCS risk score categories

Death rate increased with MC also in CVD− (n = 733; p < 0.0001). At follow-up 43 subjects (5.9%) died: 2.2% in no-MC (n = 9; 1.95 × 1000 PYs), 6.4% in 1 MC (n = 15; 5.95 × 1000 PYs), 12.9% in 2 MC (n = 8; 11.30 × 1000 PYs), and 64.7% in 3 MC (n = 11; 72.00 × 1000 PYs; Additional file 1: Figure S2). In CVD−, after adjustments (Fig. 3, Models 1 to 3), the effects of MC on all-cause mortality remained significant up to Model 3, where the HR for 3 MC was 5.46 (95% CI 1.67–17.86, p = 0.005) independently of uric acid and the EURODIAB PCS score.

### Microvascular complications and incidence of major cardiovascular and coronary events in the whole cohort

**Fig. 4:**
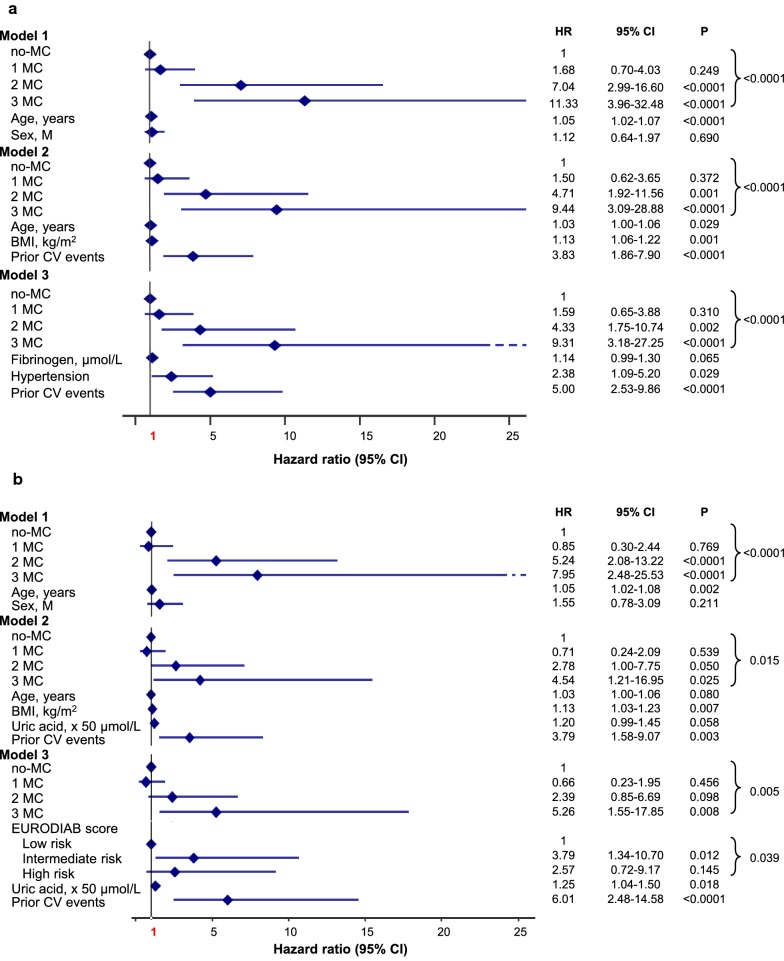
Adjusted HR for major vascular (**a**) and coronary events (**b**) by MC in the whole cohort. Model 1: adjusted for age and sex. Model 2: adjusted for age, sex, smoking habits, age at diagnosis of diabetes ≤ 18 years (n = 322, 43.8%), diabetes duration, BMI, HbA1c, LDL-cholesterol, HDL-cholesterol, triglycerides, uric acid, fibrinogen, hypertension and prior CV events. Model 3: adjusted for sex, smoking habits, age at diagnosis of diabetes ≤ 18 years, diabetes duration, LDL-cholesterol, triglycerides, uric acid, fibrinogen, hypertension, previous CV events and EURODIAB PCS risk score categories

Major CV events occurred in 49 out of 736 subjects (6.7%; 6.42 × 1000 PYs) during a mean follow-up of 10.4 ± 2.9 years. Incidence of major CV events increased with MC (p < 0.0001): no-MC 2.2% (n = 9; 2.09 × 1000 PYs; Ref), 1 MC 5.0% (n = 12; 4.79 × 1000 PYs; HR 2.27 [95% CI 0.96–5.38]), 2 MC 26.8% (n = 19; 27.56 × 1000 PYs; HR 12.88 [95% CI 5.82–28.50]), 3 MC 40.9% (n = 9; 63.82 × 1000 PYs; HR 29.34 [95% CI 11.59–74.25]; Fig. 1b). In adjusted Cox regression analysis (Fig. 4a, Models 1 to 3), the risk for major CV events, compared to no-MC, increased in subjects with 2 MC and more so in those with 3 MC. In particular, in Model 3, HRs were: 1 MC 1.59 (95% CI 0.65–3.88), 2 MC 4.33 (95% CI 1.75–10.74), 3 MC 9.31 (95% CI 3.18–27.25, p < 0.0001) with independent effects for fibrinogen, hypertension, and prior CV events.

Coronary events occurred in 35 out of 736 subjects (4.8%; 4.54 × 1000 PYs) during a mean follow-up of 10.5 ± 2.7 years with incidence increasing with MC (p < 0.0001): no-MC 2.2% (n = 9; 2.09 × 1000 PYs; Ref), 1 MC 2.5% (n = 6; 2.37 × 1000 PYs; HR 1.12 [95% CI 0.40–3.15]), 2 MC 19.7% (n = 14; 19.62 × 1000 PYs; HR 9.12 [95% CI 3.94–21.10]), 3 MC 27.3% (n = 6; 39.13 × 1000 PYs; HR 18.35 [95% CI 6.51–51.76]; Fig. 1c). In the adjusted Cox regression analysis (Fig. 4b, Models 1 to 3), the risk for coronary events, compared to no-MC, increased in subjects with 2 MC and to a greater extent in those with 3 MC. In particular, in Model 3, HRs were: 1 MC 0.66 (95% CI 0.23–1.95), 2 MC 2.39 (95% CI 0.85–6.69), 3 MC 5.26 (95% CI 1.55–17.85, p = 0.005) with independent effects for uric acid, EURODIAB PCS score and prior CV events.

### Microvascular complications and incidence of major cardiovascular and coronary events in CVD−

**Fig. 5:**
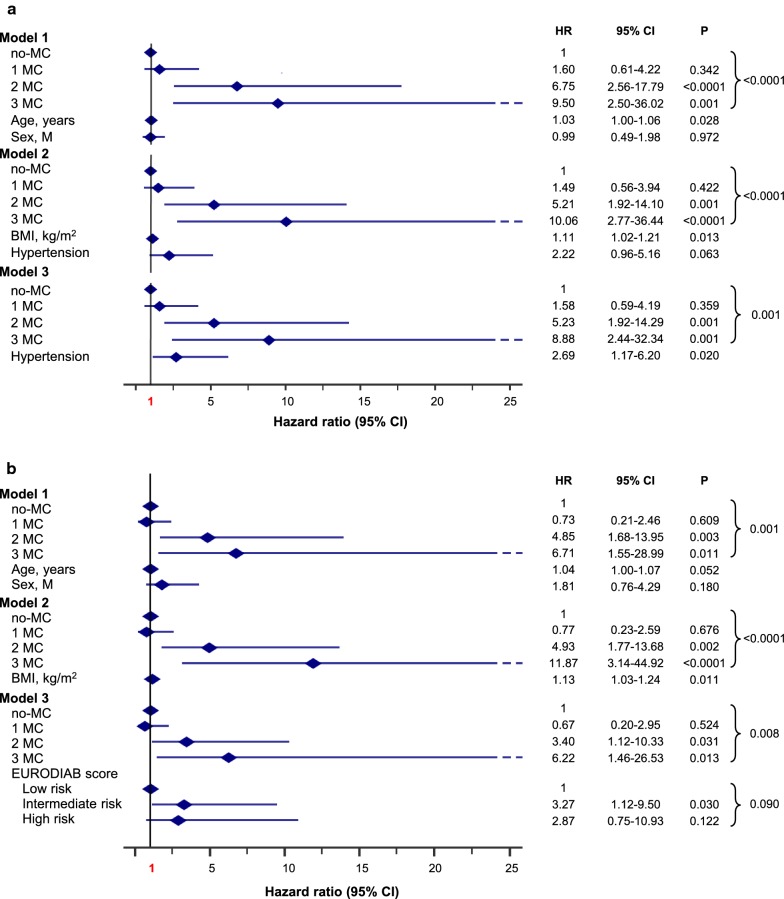
Adjusted HR for major vascular (**a**) and coronary events (**b**) by MC in CVD−. Model 1: adjusted for age and sex. Model 2: adjusted for age, sex, smoking habits, age at diagnosis of diabetes ≤ 18 years (n = 305, 43.8%), diabetes duration, BMI, HbA1c, LDL-cholesterol, HDL-cholesterol, triglycerides, uric acid, fibrinogen, hypertension and prior CV events. Model 3: adjusted for sex, smoking habits, age at diagnosis of diabetes ≤ 18 years, diabetes duration, LDL-cholesterol, triglycerides, uric acid, fibrinogen, hypertension, previous CV events and EURODIAB PCS risk score categories

Among CVD− (n = 697), incidence of major CV events (n = 32, 4.6%) increased with MC (p < 0.0001): 2.0% in no-MC (n = 8; 1.88 × 1000 PYs), 3.9% in 1 MC (n = 9; 3.77 × 1000 PYs), 18.6% in 2 MC (n = 11; 18.50 × 1000 PYs), and 26.7% in 3 MC (n = 4; 32.50 × 1000 PYs; Additional file 1: Figure S3). The effect, though attenuated, remained significant after multiple adjustments up to Model 3, where the HR for 3 MC resulted 8.88 (95% CI 2.44–32.34, p = 0.001; Fig. 5a, Model 1 to 3), with independent effect for hypertension.

Consistently, incidence of coronary events (n = 23, 3.3%) increased with MC (p < 0.0001): 2.0% in no-MC (n = 8; 1.88 × 1000 PYs), 1.8% in 1 MC (n = 4; 1.66 × 1000 PYs), 13.6% in 2 MC (n = 8; 13.24 × 1000 PYs), and 20.0% in 3 MC (n = 3; 23.47 × 1000 PYs; Additional file 1: Figure S4). Once again, the effect remained significant even after multiple adjustments (Fig. 5b, Model 1 to 3).

### Retinopathy, mortality and other outcomes

Diabetic retinopathy was the most frequent MC (n = 322; 41.6%); 82 individuals had nephropathy (10.6%) and 68 neuropathy (8.8%). The prevalence of advanced retinopathy increased from individuals with 1 to those with 3 MC (p < 0.0001; Additional file 1: Figure S1).

Mortality was 2.7% (n = 12; 2.41 × 1000 PYs; Ref) in 452 subjects without DR (DR−) and increased to 13.0% (n = 42; 12.36 × 1000 PYs, HR 5.18 [95% CI 2.72–9.83]) in the remaining 322 DR+ individuals (p < 0.0001; Additional file 1: Figure S5A). Adjustment for nephropathy and neuropathy did not reduce the risk for mortality associated with DR+ (HR 2.71 [95% CI 1.35–5.43]; p = 0.005) nor it did adjustment for other potential confounders (Additional file 1: Table S3, Models 1 to 3). In Model 3, death HRs were 2.07 (95% CI 1.02–4.19; p = 0.044) for DR+, and 3.02 (95% CI 1.24–7.36) and 9.65 (95% CI 4.02–23.16), respectively, for intermediate- and high-risk EURODIAB PCS score (p < 0.0001), with an independent role for nephropathy, smoking, and uric acid. Mortality occurred in 9.4% among subjects with non-advanced DR (n = 19 out of 202; 8.91 × 1000 PYs; HR 3.74 [95% CI 1.81–7.70]) versus 19.2% in those with advanced DR (n = 23 out of 120; 18.16 × 1000 PYs; HR 7.60 [95% CI 3.78–15.28]; p < 0.0001; Additional file 1: Figure S5B). HRs remained significant with no difference between non-advanced and advanced DR (2.71 [95% CI 1.30–5.69] and 2.69 [95% CI 1.20–6.05]; p = 0.008 and p = 0.017, respectively) after adjustment for nephropathy and neuropathy. Mortality HRs remained significant for non-advanced DR after adjustment for confounders, with an independent effects for active smoking, uric acid, and EURODIAB PCS score (data not shown).

Cumulative incidence of major CV events was 2.1% (n = 9; 1.97 × 1000 PYs; Ref) among 426 DR− and 12.9% (n = 40; 13.05 × 1000 PYs; HR 6.59 [95% CI 3.20–13.59]) in 310 DR+ (p < 0.0001; Additional file 1: Figure S6A). In this case too, the risk remained significantly higher after adjustment for nephropathy and neuropathy, and several other confounders (Additional file 1: Table S4, Models 1 to 3). In Model 3, HRs for CV events were 2.76 (95% CI 1.25–6.07; p = 0.012) for DR+, and 2.31 (95% CI 0.98–5.41) and 3.71 (95% CI 1.47–9.37) for intermediate- and high-risk EURODIAB PCS score, respectively (p = 0.021), with an independent role for nephropathy and prior CV events. Moreover, the event rate was lower for non-advanced as compared to advanced DR (p < 0.0001; Additional file 1: Figure S6B), even after adjustment for the other MC and confounders (data not shown). A similar trend was observed as far incidence of coronary events (Additional file 1: Figures S7A, B), although in Model 2 and 3 DR did not enter as a covariate of coronary events, while independent roles were played by nephropathy, prior CV disease, uric acid and EURODIAB score (Additional file 1: Table S5, Models 1 to 3).

For each outcome, in all regression models no interaction (p ≥ 0.368 for all, data not shown) has been observed between nephropathy or neuropathy with the presence of retinopathy even if, as anticipated by the low number of individuals with nephropathy or neuropathy free from retinopathy, confidence intervals should, in many cases, interpreted with caution.

## Discussion

We found that in a cohort of 774 subjects with type 1 diabetes, over a more than 10 year follow-up the presence of microvascular disease at study entry was an independent determinant of all-cause mortality and cardiovascular events. Moreover, we found that number and severity of microvascular complications were associated with an increase in the rate of all-cause death, first major cardiovascular and first coronary event. The presence of one or more MC was also associated with worse profile of conventional and non conventional CV risk factors, including a contemporary specific vascular risk score. Yet, even accounting for these variables, MC remained an independent predictor of major outcomes, including all-cause mortality and CV events. These observations were fully confirmed in the 733 individuals free of established CV disease at baseline. Finally, retinopathy, the most frequent MC in our cohort, conferred by itself a higher risk of mortality and CV outcomes irrespective of CV risk profile and of the effect of nephropathy and neuropathy.

The pathophysiologic processes leading to CV disease in type 1 diabetes are complex and largely unidentified. Microvascular beds are the sites where the earliest consequences of inflammatory processes occur, leading to endothelial and smooth muscle cell dysfunction, and altered structure and composition of the extracellular matrix. These modifications are recognized as risk factors for the development of atherosclerotic disease in large arteries as well [30]. Also, a rule in diabetic vascular complications has been suggested for advanced glycation end-products assessed by skin autofluorescence [31, 32], HDL dysfunction [33], and altered regulation of extracellular matrix remodeling [34], both in type 1 and type 2 diabetes. Among microvascular beds, the eye and the kidney are vulnerable target end-organs where functional and structural microvascular alterations anticipate and predict incident CV events [30].

In line with all this, recent cross-sectional studies reported an association between microvascular complications, mainly diabetic retinopathy, and subclinical atherosclerosis in subjects with type 1 diabetes. In a cohort of subjects with type 1 diabetes without previous CV disease or DKD, Carbonell et al. found an independent association between PDR and higher atherosclerotic burden in the carotid arteries [35]. Furthermore, in subjects with long-lasting diabetes (≥ 50 years) Lovshin et al. found that retinopathy and large-nerve fiber neuropathy were associated with presence of higher coronary artery calcification volume score [36].

These observations suggest that involvement of microvascular beds may reflect underlying, as jet unidentified, pathways linked to vascular disease.

An association between microvascular complications and risk of CV disease has been recently reported in type 2 diabetes. A cohort of 49,027 individuals with type 2 diabetes and no history of CV disease at baseline was studied by Brownrigg et al. over a median follow-up of 5.5 years [37]. In this study, the investigators found a dose-dependent relationship between the number of microvascular complications, the overall risk of experiencing a CV event, as well as risk of other endpoints such as all-cause mortality, CV death and hospitalization for heart failure. The same study found that isolated retinopathy, peripheral neuropathy or nephropathy, independent of conventional risk factors, conferred risk of CV events similar to the one associated with blood pressure, HbA_1c_ and LDL-cholesterol. Furthermore, no deviation from linearity was found in subgroups stratified by varying degrees of risk factors control [37].

On the contrary, robust data assessing the effects of microvascular complications on all-cause mortality and CV risk are still lacking in type 1 diabetes [38]. Previous reports have shown an increase in CV risk and mortality in subjects with type 1 diabetes with diabetic kidney disease [9, 12, 39–42] and peripheral [43] or autonomic neuropathy [43–45]. A recent report from FinnDiane showed that in subjects with long-standing type 1 diabetes severe DR, even without DKD, increases the risk of CV disease, in particular for peripheral artery disease, independently of common CV risk factors [46].

We now provide evidence that incidence of all-cause death increases in individuals with type 1 diabetes with one (6.76/1000 PYs), two (12.93/1000 PYs) or three (79.77/1000 PYs) as compared to subjects free of microvessel damage (1.92/1000 PYs). Adjustment for potential confounders attenuated HR for primary outcome across all three groups, but the microvascular burden remained an independent predictor in particular for those individuals with 3 MC. This was also true among those subjects without prior CV events even after correction for the EURODIAB PCS score, a powerful integrated predictor of major outcomes including all-cause mortality. The association is similar, if not stronger, between cumulative burden of MC and major vascular events. Unadjusted rates were 2.09, 4.79, 27.56, and 63.82/1000 PYs for individuals with no-MC and for those with one, two, or three MC, respectively. Again HRs for major vascular outcomes were attenuated across the three groups after adjustment for confounders. A similar pattern was observed for coronary events.

The attenuation of the HRs for each outcome after multiple adjustments confirms the role of conventional CV risk factors. Yet, the persistence of the predictive effect of MC, even after full adjustment (i.e. Model 3), supports the negative impact of existing microvascular complications, which exert an effect similar to the one of active smoking on all-cause mortality (Fig. 2) [47]. Also, the coexistence of two microvascular complications is a stronger predictor for major CV events than hypertension (Fig. 4a) and three MC are associated with coronary events to a greater extent than the EURODIAB PCS score (Fig. 4b).

We found no independent association of baseline HbA_1c_ with mortality, major vascular outcomes or coronary events. Besides the much debated effect of glycaemic control on these outcomes, the present finding should be interpreted with caution as a single baseline HbA_1c_ value is unlikely to capture and predict cumulative exposure to hyperglycemia. Also, one could argue that glycaemic control is well incorporated in the degree of the microvascular bed involvement given the well known relationship between glucose control and development of microvessel damage.

Due to the size of our study population, we could not analyze data according to individual effect of retinopathy, nephropathy or peripheral neuropathy. However, we found that retinopathy, the most common microvascular complication in our cohort, conferred per se an increased risk for all-cause mortality and vascular outcomes, an association that remained valid after adjustment for concomitant nephropathy and peripheral neuropathy as well as adjustment for other confounders.

This particular finding is at variance with the results very recently published by Bjerg et al. [15]. In a larger population of 3828 subjects with type 1 diabetes over a 26,665 PYs follow-up they found that neuropathy and diabetic kidney disease were strong and independent risk factors of mortality with no evidence for an association with retinopathy. Furthermore, on the contrary of the present study, authors found no evidence that multiple complications could increase the risk conferred by each complication separately [15]. A number of reasons could account for these discrepancies, including difference in population size. Since diabetic retinopathy was by far the most common microvascular condition in a smaller population with a longer follow-up, this may have greater chance to show a significant association. Nonetheless, the relative role of microvascular complications on mortality and cardiovascular risk in type 1 diabetes may require more attention. Systematic reviews have recently examined the associations between diabetic retinopathy and common macrovascular complications [48, 49]. The analysis by Pearce et al. included both type 1 and type 2 diabetes and showed that DR was consistently associated with other complications of diabetes. In keeping with our own results, the meta-analysis also reported an association between severity of DR and CV risk. In particular, DR was a strong predictor of stroke, major CV events and peripheral artery disease [48]. In the meta-analysis by Guo et al. DR was associated with increased risk of CV disease (relative risk 2.42 [95% CI 1.77–3.31]); this risk was stronger in type 1 diabetes (relative risk 3.59 [95% CI 1.79–7.20]) [49]. A recent report from FinnDiane found that severe DR, even in the absence of concomitant DKD, increases cardiovascular risk, in particular the risk of peripheral artery disease, independently of common CV risk factors [46]. Moreover, the Joslin Medalist Study showed that the CV risk was greater in subjects with severe DR on top of DKD than in those with DKD alone [14]. In summary, besides the discrepancy with respect to the specific effect of DR, our study and the one by Bjerg et al. [15] support the concept that microvascular complications can contribute in increasing the risk of all-cause and cardiovascular mortality in subjects with type 1 diabetes.

There are a number of limitations of the present study. First, the number of events (54 deaths and 49 major CV events) was limited suggesting some caution should be used in extrapolating from multivariate analysis and its clinical implications. Second, we have no information on the course of microvascular diseases over time; this precluded us to assess the effects of progression or regression of the microvascular involvement on relevant outcomes. For instance, while progression of diabetic nephropathy confers an increased risk for cardiovascular disease and premature death, it has been recently reported that, in type 1 diabetes, regression of albuminuria reduces the risk of cardiovascular events and all-cause and cardiovascular mortality to the same level as for those who did not progress [50]. A similar limitation of the study is the lack of data on time-varying exposure to glycated haemoglobin levels and conventional cardiovascular risk factors during follow-up. Third, data on autonomic neuropathy were not available at baseline. In the Diabetes Control and Complications Trial/Epidemiology of Diabetes Intervention and Complications (DCCT/EDIC) cohort, individuals with cardiovascular autonomic neuropathy at study closeout had higher long-term risk of CV events [45]. A further limitation of our study is that we could not retrieve reliable data about cause of death, including CV death, from the Regional Registry, somewhat limiting the detail of study results. Finally, the results of this study should be interpreted taking into consideration the characteristics of our cohort, with retinopathy being by far the most frequent microvascular complication. Thus, in addition to the co-existence of microvascular complications, also the severity of retinopathy (as an alternative way to assess the degree of microvascular burden) has been associated with each study outcome.

The study, however, also have some strengths, such as the single-centre nature of the study and the relatively long follow-up. Moreover, registry linkage with individuals’ unique personal identification codes provides survival information for virtually all participants and 95% coverage for major vascular events. Of relevance, details on retinopathy classification was available for all individuals allowing meaningful sensitivity analyses for advanced and early stages of the condition.

## Conclusions

In summary, our data corroborate initial information and more recent publications [15] that the presence and severity of microvascular complications contribute in an independent manner to increase the risk of all-cause mortality and cardiovascular outcomes in individuals with type 1 diabetes. As the assessment of microvascular disease is an essential component of routine clinical care, these findings show how screening and staging for microvascular complications can offer a convenient and unexpensive tool for improving risk prediction in subjects with type 1 diabetes.

## Supplementary information

**Additional file 1: Table S1.** International Classification of Diseases (ICD-9) system codes collected during follow-up. **Table S2.** Baseline clinical characteristics of individuals with retinopathy, nephropathy and neuropathy compared with those with no MC. **Table S3.** Survival analysis by Cox proportional hazards regression according to presence of any degree of retinopathy at baseline. **Table S4.** Incidence analysis of major CV events by Cox proportional hazards regression according to presence of any degree of retinopathy at baseline. **Table S5.** Incidence analysis of coronary events by Cox proportional hazards regression according to presence of any degree of retinopathy at baseline. **Figure S1.** Distribution of microvascular diseases in the 774 participants with type 1 diabetes. **Figure S2.** Kaplan–Meier curves showing survival from all-cause death by MC groups in individuals without prior CV events. **Figure S3**. Kaplan–Meier curves showing cumulative incidence of major vascular events by MC groups in individuals without prior CV events. **Figure S4.** Kaplan–Meier curves showing cumulative incidence of coronary events in by MC groups in individuals without prior CV events. **Figure S5.** Kaplan–Meier curves showing survival from all-cause death in participants with retinopathy vs. those without DR. **Figure S6.** Kaplan–Meier curves showing cumulative incidence of major CV events in participants with retinopathy vs. those without DR. **Figure S7.** Kaplan–Meier curves showing cumulative incidence of coronary events in participants with retinopathy vs. those without DR.

## Data Availability

Data collected for this study can be shared and made available upon reasonable request to the corresponding author and subject to an approved proposal and data access agreement.

## Abbreviations

ACR: albumin-to-creatinine ratio
ALT: alanine aminotransferase
AST: aspartate aminotransferase
BMI: body mass index
BP: blood pressure
CHD: coronary artery disease
CI: confidence intervals
CKD-EPI: chronic kidney disease epidemiology collaboration equation
CV: cardiovascular
CVD: cardiovascular disease
DD: diabetes duration
DKD: diabetic kidney disease
DR: diabetic retinopathy
EURODIAB PCS: EURODIAB Prospective Complications Study
eGFR: estimated glomerular filtration rate
GGT: gamma-glutamyltransferase
HR: hazard ratio
HS: high score
IS: intermediate score
K–M: Kaplan–Meier
LE: life expectancy
LS: low score
MC: microvascular complication
PDR: proliferative diabetic retinopathy
PYs: person-years
RAS: renin angiotensin system
WHR: waist-to-hip ratio
