# Supplementary material for: Microvascular complications burden (nephropathy, retinopathy and peripheral polyneuropathy) affects risk of major vascular events and all-cause mortality in type 1 diabetes: a 10-year follow-up study
Source: Cardiovasc Diabetol. 2019 Nov 16;18:159. doi: 10.1186/s12933-019-0961-7 (PMC6858978; doi:10.1186/s12933-019-0961-7)
Supplement: Supplementary file 1 — Additional file 1: Table S1. International Classification of Diseases (ICD-9) system codes collected during follow-up. Table S2. Baseline clinical characteristics of individuals with retinopathy, nephropathy and neuropathy compared with those with no MC. Table S3. Survival analysis by Cox proportional hazards regression according to presence of any degree of retinopathy at baseline. Table S4. Incidence analysis of major CV events by Cox proportional hazards regression according to presence of any degree of retinopathy at baseline. Table S5. Incidence analysis of coronary events by Cox proportional hazards regression according to presence of any degree of retinopathy at baseline. Figure S1. Distribution of microvascular diseases in the 774 participants with type 1 diabetes. Figure S2. Kaplan–Meier curves showing survival from all-cause death by MC groups in individuals without prior CV events. Figure S3. Kaplan–Meier curves showing cumulative incidence of major vascular events by MC groups in individuals without prior CV events. Figure S4. Kaplan–Meier curves showing cumulative incidence of coronary events in by MC groups in individuals without prior CV events. Figure S5. Kaplan–Meier curves showing survival from all-cause death in participants with retinopathy vs. those without DR. Figure S6. Kaplan–Meier curves showing cumulative incidence of major CV events in participants with retinopathy vs. those without DR. Figure S7. Kaplan–Meier curves showing cumulative incidence of coronary events in participants with retinopathy vs. those without DR. [file 12933_2019_961_MOESM1_ESM.doc]

Microvascular complications burden (nephropathy, retinopathy and peripheral polyneuropathy) affects risk of major vascular events and all-cause mortality in type 1 diabetes: A 10-year follow-up study

*Monia Garofolo^1^ MD, Elisa Gualdani^2^ BSc, Rosa Giannarelli^1^ MD, Michele Aragona^1^ MD, Fabrizio Campi^1^ MD, Daniela Lucchesi^1^ MSc, Giuseppe Daniele^1^ PhD, Roberto Miccoli^1^ PhD, Paolo Francesconi^2^ MD, Prof Stefano Del Prato^1^, Prof Giuseppe Penno^1^*

^1^ Section of Diabetes and Metabolic Disease, Department of Clinical and Experimental Medicine, University of Pisa and Azienda Ospedaliero-Universitaria Pisana, Pisa, Italy

^2^ Epidemiology Unit, Regional Healthcare Agency of Tuscany, Florence, Italy.

**Additional Table S1 – International Classification of Diseases (ICD-9) system codes collected during follow-up.**

| Coronary heart disease including acute myocardial infarction | ICD-9 410-414 |
| --- | --- |
| Coronary revascularization | ICD-9 00.66, 36.0X, 36.1X |
| Stroke | ICD-9 431, 432.X, 433, 434, 436, 437.X |
| Carotid revascularization | ICD-9 00.61-00.65, 38.02, 38.12, 39.74 |
| Ulcer, gangrene and amputation | ICD-9 440.23, 440.24, 84.1 |
| Lower limb revascularization | ICD-9 38.18, 39.25, 39.50, 39.90 |
| Intervention for aneurysm of the aorta | ICD-9 441.X, 38.04, 38.64, 39.71 |

The Inpatient Registry includes mandatory information on all principal and secondary hospital discharge diagnoses.

**Additional Table S2. Baseline clinical characteristics of individuals with retinopathy, nephropathy and neuropathy compared with those with no MC.**

|  | No MC  (n=425) | Retinopathy  (n=322) | p value | Nephropathy  (n=82) | p value | Neuropathy  (n=68) | p value |
| --- | --- | --- | --- | --- | --- | --- | --- |
| Men/women | 224/201 (52·7%/47·3%) | 168/154  (52·2%/47·8%) | 0·885 | 49/33  (59·8%/40·2%) | 0·241 | 36/32  (47·1%/52·9%) | 0·971 |
| Age (years) | 36·3 (10·1) | 45·2 (11·7) | <0·0001 | 46·9 (14·3) | <0·0001 | 53·0 (12·4) | <0·0001 |
| Age at diabetes diagnosis (years) | 23·2 (10·7) | 17·5 (10·2) | <0·0001 | 20·4 (11·6) | 0·401 | 21·8 (11·0) | 0·341 |
| Duration of diabetes (years) | 13·1 (9·4) | 27·7 (10·6) | <0·0001 | 26·5 (12·1) | <0·0001 | 31·2 (12·6) | <0·0001 |
| BMI (kg/m^2^) | 24·2 (3·3) | 25·6 (3·7) | <0·0001 | 26·2 (4·44) | <0·0001 | 26·0 (3·3) | <0·0001 |
| WHR | 0·914 (0·056) | 0·929 (0·071) | 0·003 | 0·941 (0·077) | 0·004 | 0·946 (0·066) | <0·0001 |
| Smoking habits: non-smokers, current smokers (n=496) | 295/121  (70·9%/29·1%) | 226/90  (71·5%/28·5%) | 0·858 | 54/26  (67·5%/32·5%) | 0·540 | 48/19  (71·6%/28·4%) | 0·903 |
| Fasting glucose (mmol/L) | 9·10 (4·23) | 9·71 (4·92) | 0·078 | 11·3 (5·03) | <0·0001 | 10·86 (4·75) | 0·002 |
| HbA_1c_ (% and mmol/mol) | 7·70 (1·19)  60·6 (13·0) | 7·98 (1·12)  63·7 (12·2) | <0·0001 | 8·36 (1·24)  67·8 (13·5) | <0·0001 | 8·30 (1·25)  67·2 (13·6) | <0·0001 |
| Systolic BP (mm Hg) | 122 (15) | 133 (19) | <0·0001 | 140 (20) | <0·0001 | 140 (19) | <0·0001 |
| Diastolic BP (mm Hg) | 72 (8) | 75 (9) | <0.0001 | 77 (10) | <0.0001 | 75 (10) | 0·027 |
| Total cholesterol (mmol/L) | 4·72 (0·85) | 4·96 (0·86) | <0·0001 | 5·14 (1·11) | 0·002 | 4·86 (0·88) | 0·237 |
| LDL cholesterol (mmol/L) | 2·94 (0·76) | 3·07 (0·72) | 0·014 | 3·21 (0·91) | 0·004 | 2·97 (0·80) | 0·738 |
| HDL cholesterol (mmol/L)  Men  Women | 1·44 (1·22–1·71)  1·71 (1·42–1·99) | 1·45 (1·24–1·68)  1·79 (1·55–2·13) | 0·823  0·038 | 1·53 (1·29–1·77)  1·76 (1·42–2·07) | 0·734  0·711 | 1·45 (1·30–1·68)  1·77 (1·59–1·99) | 0·455  0·519 |
| Triacylglycerol (mmol/L)  Men  Women | 0·86 (0·69–1·15)  0·75 (0·58–1·04) | 0·95 (0·74–1·42)  0·81 (0·63–1·06) | 0·027  0·645 | 1·12 (0·83–1·75)  0·99 (0·72–1·32) | 0·003  0·012 | 1·13 (0·80–1·56)  0·86 (0·69–1·15) | 0·025  0·171 |
| ALT (U/L) | 19·7 (11·5) | 20·2 (9·9) | 0·504 | 22·3 (13·2) | 0·068 | 21·7 (12·9) | 0·175 |
| AST (U/L) | 20·9 (16·1) | 23·7 (45·4) | 0·286 | 24·0 (23·4) | 0·146 | 24·3 (24·7) | 0·138 |
| Gamma-GT (U/L) | 18·4 (27·9) | 22·6 (38·5) | 0·108 | 33·6 (70·9) | 0·061 | 36·3 (80·0) | 0·072 |
| Uric acid (µmol/L) | 213·7 (58·3) | 235·2 (74·5) | <0·0001 | 277·4 (106·4) | <0·0001 | 257·2 (97·1) | 0·001 |
| Fibrinogen (µmol/L) | 9·63 (1·93) | 10·21 (1·99) | <0·0001 | 11·10 (2·26) | <0·0001 | 10·70 (1·98) | <0·0001 |
| Creatinine (µmol/L) | 70·8 (12·8) | 76·7 (23·9) | <0·0001 | 91·7 (39·4) | <0·0001 | 84·2 (39·8) | 0·008 |
| Albumin-to-creatinine ratio (mg/mmol) | 0·40  (0·23–0·75) | 0·60  (0·29–1·50) | <0·0001 | 7·50  (3·55–24·23) | <0·0001 | 0·80  (0·37–5·92) | 0·001 |
| eGFR, CKD-EPI  (mL/min per 1.73 m^2^) | 107·4 (14·1) | 96·0 (19·0) | <0·0001 | 85·6 (28·5) | <0·0001 | 86·4 (24·1) | <0·0001 |
| Daily insulin dose  (IU/kg body weight) | 0·66 (0·21) | 0·67 (0·20) | 0·298 | 0·71 (0·20) | 0·787 | 0·68 (0·24) | 0·418 |
| MDI/CSII | 380/45  (89·4%/10·6%) | 286/36  (88·8%/11·2%) | 0·797 | 77/5  (93·9%/6·1%) | 0·212 | 59/9  (86·5%/13·2%) | 0·516 |
| Treatment with BP-lowering agents | 28 (6·6%) | 116 (36·0%) | <0·0001 | 52 (63·4%) | <0·0001 | 33 (48·5%) | <0·0001 |
| Treatment with RAS blockers | 23 (5·4%) | 108 (33·5%) | <0·0001 | 49 (59·8%) | <0·0001 | 28 (41·2%) | <0·0001 |
| Treatment with lipid-lowering agents | 35 (8·2%) | 59 (18·3%) | <0·0001 | 21 (25·6%) | <0·0001 | 21 (30·9%) | <0·0001 |
| Treatment with antiplatelet drugs | 6 (1·4%) | 42 (13·0%) | <0·0001 | 49 (59·8) | <0·0001 | 18 (26·5) | <0·0001 |
| Treatment with metformin | 18 (4·2%) | 28 (8·7%) | 0·012 | 5 (6·1%) | 0·458 | 6 (8·8%) | 0·103 |
| Hypertension | 82 (19·3%) | 176 (54·7%) | <0·0001 | 64 (78·0%) | <0·0001 | 49 (72·1) | <0·0001 |
| Retinopathy: no retinopathy/non advanced/advanced | 425/0/0  (100%/0/0) | 0/202/120  (0/62·7%/37·3%) | --- | 18/20/44  (22·0%/24·4%/53·7%) | <0·0001 | 10/24/34  (14·7%/35·3%/50·0%) | <0·0001 |
| Peripheral polyneuropathy | 0  (0) | 58 (18·0%) | <0·0001 | 25 (30·5%) | <0·0001 | 68 (100) | --- |
| Diabetic kidney disease | 0 (0) | 64 (19·9%) | <0·0001 | 82 (100) | --- | 25 (63·2%) | <0·0001 |
| Major adverse cardiovascular events (MACE) | 7 (1·6%) | 33 (10·2%) | <0·0001 | 12 (14·6%) | <0·0001 | 16 (23·5%) | <0·0001 |
| Coronary artery disease | 3 (0·7%) | 25 (7·8%) | <0·0001 | 10 (12·2%) | <0·0001 | 13 (19·1%) | <0·0001 |
| Stroke | 2 (0·5%) | 2 (0·6%) | 1·000 | 1 (1·2%) | 0·412 | 1 (1·5%) | 0·360 |
| EURODIAB PCS risk score: LS, IS, HS | 324/84/17  (76·2%/19·8%/4·0%) | 133/111/78  (41·3%/34·5%/24·2%) | <0·0001 | 17/25/40  (20·7%/30·5%/48·8%) | <0·0001 | 8/23/37  (11·8%/33·8%/54·4%) | <0·0001 |
| Cancer | 3 (0·7%) | 6 (1·9%) | 0·151 | 2 (2·4%) | 0·146 | 3 (4·4%) | 0·037 |
| Autoimmune thyreopathy | 58 (13·6%) | 49 (15·2%) | 0·544 | 10 (12·2%) | 0·724 | 10 (14·7%) | 0·814 |

Data are n (%) or mean (SD) or median (interquartile range)

MDI/CSII, Multiple Daily Insulin Injections/Continuous Subcutaneous Insulin Infusion

LS, low score; IS, intermediate score; HS, high score

**Additional Table S3** - **Survival analysis by Cox proportional hazards regression according to presence of any degree of retinopathy at baseline.**

|  | **HR** | **95% CI** | **p** |
| --- | --- | --- | --- |
| **Preliminary adjustment** |  |  |  |
| Retinopathy |  |  |  |
| No retinopathy | 1 | – |  |
| No advanced/Advanced | 2·71 | 1·35–5·43 | 0·005 |
| Nephropathy | 4·63 | 2·60–8·25 | <0·0001 |
| Peripheral neuropathy | 2·65 | 1·46–4·80 | 0·001 |
| **Model 1** |  |  |  |
| Age, x 1 year | 1·07 | 1·04–1·09 | <0·0001 |
| Sex, men | 1·81 | 1·03–3·19 | 0·039 |
| Retinopathy |  |  |  |
| No retinopathy | 1 | – |  |
| No advanced/Advanced | 1·93 | 0·95–3·92 | 0·068 |
| Nephropathy | 4·20 | 2·37–7·46 | <0·0001 |
| Other variables included in the model, but not selected as independent variables: peripheral neuropathy | | | |
| **Model 2** |  |  |  |
| Age, x 1 year | 1·07 | 1·04–1·09 | <0·0001 |
| Active smoking | 2·41 | 1·28–4·53 | 0·006 |
| Triglycerides, x 0.1 mmol/L | 1·03 | 1·00–1·06 | 0·033 |
| Uric acid, x 50 μmol/L | 1·24 | 1·08–1·43 | 0·003 |
| Retinopathy |  |  |  |
| No retinopathy | 1 | – |  |
| No advanced/Advanced | 2·16 | 1·07–4·38 | 0·032 |
| Nephropathy | 2·55 | 1·36–4·77 | 0·003 |
| Other variables included in the model, but not selected as independent variables: sex, diabetes duration, BMI, HbA1c, LDL-cholesterol, HDL-cholesterol, fibrinogen, peripheral neuropathy, hypertension and prior CV events | | | |
| **Model 3** |  |  |  |
| Active smoking | 1·94 | 1·07–3·52 | 0·029 |
| Uric acid, x 50 μmol/L | 1·25 | 1·06–1·39 | 0·005 |
| EURODIAB PCS risk score |  |  | <0·0001 |
| LS | 1 | – |  |
| IS | 3·02 | 1·24–7·36 | 0·015 |
| HS | 9·65 | 4·02–23·16 | <0·0001 |
| Retinopathy |  |  |  |
| No retinopathy | 1 | – |  |
| No advanced/Advanced | 2·07 | 1·02–4·19 | 0·044 |
| Nephropathy | 2·06 | 1·08–3·94 | 0·028 |
| Other variables included in the model, but not selected as independent variables: sex, diabetes duration, LDL-cholesterol, triglycerides, fibrinogen, peripheral neuropathy, hypertension and prior CV events | | | |

LS, low score; IS, intermediate score; HS, high score

Data are preliminary adjusted for nephropathy and peripheral neuropathy and then adjusted for age and sex (Model 1), for multiple potential confounders (Model 2) and for multiple confounders including EURODIAB PCS risk score (Model 3)

**Additional Table S4 - Incidence analysis of major CV events by Cox proportional hazards regression according to presence of any degree of retinopathy at baseline.**

|  | **HR** | **95% CI** | **p** |
| --- | --- | --- | --- |
| **Preliminary adjustment** |  |  |  |
| Retinopathy |  |  |  |
| No retinopathy | 1 | – |  |
| No advanced/Advanced | 3·98 | 1·85–8·54 | <0·0001 |
| Nephropathy | 4·01 | 2·18–7·37 | <0·0001 |
| Peripheral neuropathy | 2·52 | 1·34–4·76 | 0·004 |
| **Model 1** |  |  |  |
| Age, x 1 year | 1·06 | 1·04–1·08 | <0·0001 |
| Retinopathy |  |  |  |
| No retinopathy | 1 | – |  |
| No advanced/Advanced | 3·16 | 1·47–6·77 | 0·003 |
| Nephropathy | 3·71 | 2·03–6·78 | <0·0001 |
| Other variables included in the model, but not selected as independent variables: sex, peripheral neuropathy | | | |
| **Model 2** |  |  |  |
| Age, x 1 year | 1·04 | 1·01–1·07 | 0·002 |
| BMI, kg/m^2^ | 1·12 | 1·05–1·20 | 0·001 |
| Prior CV events | 4·26 | 2·13–8·54 | <0·0001 |
| Retinopathy |  |  |  |
| No retinopathy | 1 | – |  |
| No advanced/Advanced | 2·48 | 1·13–5·47 | 0·024 |
| Nephropathy | 3·21 | 1·73–5·95 | <0·0001 |
| Other variables included in the model, but not selected as independent variables: sex, active smoking, diabetes duration, HbA1c, LDL-cholesterol, HDL-cholesterol, triglycerides, uric acid, fibrinogen, peripheral neuropathy and hypertension | | | |
| **Model 3** |  |  |  |
| Prior CV events | 5·20 | 2·54–10·64 | <0·0001 |
| EURODIAB PCS risk score |  |  | 0·021 |
| LS | 1 | – |  |
| IS | 2·31 | 0·98–5·41 | 0·054 |
| HS | 3·71 | 1·47–9·37 | 0·005 |
| Retinopathy |  |  |  |
| No retinopathy | 1 | – |  |
| No advanced/Advanced | 2·76 | 1·25-6·07 | 0·012 |
| Nephropathy | 2·84 | 1·50-5·36 | 0·001 |
| Other variables included in the model, but not selected as independent variables: sex, active smoking, diabetes duration, LDL-cholesterol, triglycerides, uric acid, fibrinogen, peripheral neuropathy and hypertension | | | |

LS, low score; IS, intermediate score; HS, high score

Data are preliminary adjusted for nephropathy and peripheral neuropathy and then adjusted for age and sex (Model 1), for multiple potential confounders (Model 2) and for multiple confounders including EURODIAB PCS risk score (Model 3)

**Additional Table S5 - Incidence analysis of coronary events by Cox proportional hazards regression according to presence of any degree of retinopathy at baseline.**

|  | **HR** | **95% CI** | **p** |
| --- | --- | --- | --- |
| **Preliminary adjustment** |  |  |  |
| Retinopathy |  |  |  |
| No retinopathy | 1 | – |  |
| No advanced/Advanced | 2·48 | 1·10-5·61 | 0·029 |
| Nephropathy | 4·33 | 2·08-8·99 | <0·0001 |
| Peripheral neuropathy | 2·48 | 1·15-5·32 | 0·020 |
| **Model 1** |  |  |  |
| Age, x 1 year | 1·05 | 1·03–1·08 | <0·0001 |
| Retinopathy |  |  |  |
| No retinopathy | 1 | – |  |
| No advanced/Advanced | 2·04 | 0·91–4·59 | 0·085 |
| Nephropathy | 4·13 | 2·02–8·45 | <0·0001 |
| Other variables included in the model, but not selected as independent variables: sex, peripheral neuropathy | | | |
| **Model 2** |  |  |  |
| Age, x 1 year | 1·04 | 1·01–1·07 | 0·009 |
| BMI, kg/m^2^ | 1·12 | 1·03–1·21 | 0·008 |
| Uric acid, x 50 μmol/L | 1·18 | 0·98–1·43 | 0·088 |
| Prior CV events | 4·70 | 2·06–10·73 | <0·0001 |
| Nephropathy | 2·92 | 1·31–6·50 | 0·009 |
| Other variables included in the model, but not selected as independent variables: sex, active smoking, diabetes duration, HbA1c, LDL cholesterol, HDL cholesterol, triglycerides, fibrinogen, retinopathy, peripheral neuropathy and hypertension | | | |
| **Model 3** |  |  |  |
| Uric acid, x 50 μmol/L | 1·20 | 1·00–1·45 | 0·055 |
| Prior CV events | 7·04 | 2·93–16·94 | <0·0001 |
| EURODIAB PCS risk score |  |  | 0.022 |
| LS | 1 | – |  |
| IS | 3·97 | 1·50–10·56 | 0·006 |
| HS | 3·09 | 0·94–10·22 | 0·064 |
| Nephropathy | 2·94 | 1·29–6·68 | 0·010 |
| Other variables included in the model, but not selected as independent variables: age, sex, active smoking, diabetes duration, LDL-cholesterol, triglycerides, fibrinogen, peripheral neuropathy and hypertension | | | |

LS, low score; IS, intermediate score; HS, high score

Data are preliminary adjusted for nephropathy and peripheral neuropathy and then adjusted for age and sex (Model 1), for multiple potential confounders (Model 2) and for multiple confounders including EURODIAB PCS risk score (Model 3)

**Additional Figure S1 - Distribution of microvascular diseases in the 774 participants with type 1 diabetes.**

Out of this 774 subjects, 425 (54·9%) had no-MC, 250 (32·3%) had 1-MC, 75 (9·7%) 2-MC, and 24 (3·1%) 3-MC at baseline. Retinopathy was the most frequent MC (n=322; 41·6%); 82 individuals had nephropathy (10·6%); 68 had peripheral neuropathy (8·8%). Prevalence of advanced retinopathy increased from individuals with 1-MC (26·8%) to those with 2-MC (56·8%) and 3-MC (75·0%; p<0·0001).

**Additional Figure S2 - Kaplan-Meier curves showing survival from all-cause death by MC groups in individuals without prior CV events.**

The cohort counts now 733 subjects; out of these 43 died (5·9%). Mortality increased with increasing microvascular burden: logrank 136·35, p<0·0001. Pairwise over strata logrank statistic provides the following results: 1-MC (dashed blue line) vs. no-MC (dotted green line), logrank 7·88 (p=0·005); 2-MC (dashdotted violet line) vs. no-MC (dotted green line), logrank 15·27 (p=0·0001); furthermore, 3-MC (solid red line) vs. no-MC (dotted green line), logrank 164·06, vs. 1-MC (dashed blue line), logrank 59·58 and vs. 2-MC, logrank 22·05 (p<0·0001, for all). Percentages of death and Cox proportional unadjusted HRs (95% CI) are shown for each MC group (for overall MC, p<0·0001).

For completeness, we briefly report crude data about the 41 participants with prior CV events. Out of 41 individuals, 11 died (26·8%). Also in this small subgroup, mortality increased with increasing microvascular burden: no death in no-MC; 3 deaths in 1-MC (21·4%), 3 deaths in 2-MC (23·1%) and 5 deaths in 3-MC (71·4%): logrank 13·30, p=0·004. Pairwise over strata logrank statistic provides the following results: 3-MC vs. no-MC, logrank 5·24; vs. 1-MC, logrank 5·83 and vs. 2-MC, logrank 5·94 (p<0·05, for all). Unadjusted Cox regression, p=0·052.

**Additional Figure S3 - Kaplan-Meier curves showing cumulative incidence of major vascular events by MC groups in individuals without prior CV events.**

The cohort counts now 697 subjects; out of these 32 had major vascular events (4·6%). Cumulative incidence increased with increasing microvascular burden: logrank 54·14, p<0·0001. Pairwise over strata logrank statistic provides the following results: 2-MC (dashdotted violet line) vs. no-MC (dotted green line), logrank 36·61 (p<0·0001) and vs. 1-MC (dashed blue line), logrank 15·47 (p=0·0001); furthermore, 3-MC (solid red line) vs. no-MC (dotted green line), logrank 38·15, vs. 1-MC (dashed blue line), logrank 19·03 (p<0·0001, for both). Percentages of major vascular events and Cox proportional unadjusted HRs (95% CI) are shown for each MC group (for overall MC, p<0·0001).

For completeness, we briefly report crude data about the 39 participants with prior CV events. Out of 39 individuals, 17 had a major cardiovascular event (43·6%). Also in this subgroup incidence of major cardiovascular events increased with the microvascular burden: 1 event in no-MC (14·3%); 3 events in 1-MC (23·1%), 8 events in 2-MC (66·6%) and 5 events in 3-MC (71·4%): logrank 17·22, p=0·001. Pairwise over strata logrank statistic provides the following results: 3-MC vs. no-MC, logrank 5·68 (p=0·017), vs. 1-MC, logrank 13·18 (p<0·0001) and vs. 2-MC, logrank 6·09 (p=0·014). Unadjusted Cox regression, p=0·006.

**Additional Figure S4 - Kaplan-Meier curves showing cumulative incidence of coronary events by MC groups in individuals without prior CV events.**

The cohort counts now 697 subjects; out of these 23 had coronary events (3·3%). Cumulative incidence increased with increasing microvascular burden: logrank 38·75, p<0·0001. Pairwise over strata logrank statistic provides the following results: 2-MC (dashdotted violet line) vs. no-MC (dotted green line), logrank 20·75 (p<0·0001) and vs. 1-MC (dashed blue line), logrank 15·72 (p=0·0001); 3-MC (solid red line) vs. no-MC (dotted green line), logrank 21·70 and vs. 1-MC (dashed blue line), logrank 20·93 (p<0·0001, for both). Percentages of coronary events and Cox proportional unadjusted HRs (95% CI) are shown for each MC group (for overall MC, p<0·0001).

For completeness, we briefly report crude data about the 39 participants with prior CV events. Out of 39 individuals, 12 had a coronary event (30·8%). Also in this subgroup incidence of coronary events increased with the microvascular burden: 1 event in no-MC (14·3%); 2 events in 1-MC (15·4%), 6 events in 2-MC (50·0%) and 3 events in 3-MC (42·9%): logrank 8·14, p=0·043. Pairwise over strata logrank statistic provides the following results: 2-MC vs. 1-MC, logrank 2·82 (p=0·093); 3-MC vs. 1-MC, logrank 5·67 (p=0·017). Unadjusted Cox regression, p=0·089.

**Additional Figure S5 - Kaplan-Meier curves showing survival from all-cause death in participants with retinopathy vs. those without DR.**

Mortality was higher in DR+: logrank 31·44, p<0·0001. Panel B: within DR+, mortality was higher in subjects with advanced DR (19·2%) as compared to those with non-advanced DR (9·4%; overall logrank 41·93; p<0·0001); pairwise over strata logrank statistic provides the following results: non-advanced DR+ vs. DR-, logrank 14·54 (p=0·0001); advanced DR+ vs. DR-, logrank 44·88 (p<0·0001), and vs. non-advanced DR+, logrank 5·36 (p=0·021). Percentages of death and Cox proportional unadjusted HRs (95% CI) are shown vs. DR- (p<0·0001).

**Additional Figure S6 - Kaplan-Meier curves showing cumulative incidence of major CV events in participants with retinopathy vs. those without DR.**

Incidence rate was higher in DR+: logrank 34·83, p<0·0001. Panel B: within DR+, mortality was higher in subjects with advanced DR (20·2%; 21·17 x 1000 PYs) as compared to those with non-advanced DR (8·7%, 8·60 x 1000 PYs; overall logrank 53·41; p<0·0001); pairwise over strata logrank statistic provides the following results: non-advanced DR vs. DR-, logrank 14·84 (p=0·0001); advanced DR vs. DR-, logrank 56·79 (p<0·0001), and vs. non-advanced DR, logrank 9·55 (p=0·002). Percentages of death and Cox proportional unadjusted HRs (95% CI) are shown vs. DR- (p<0·0001).

**Additional Figure S7 - Kaplan-Meier curves showing cumulative incidence of coronary events in participants with retinopathy vs. those without DR.**

Incidence rate was higher in DR+: logrank 16·60, p<0·0001. Panel B: within DR+, mortality was higher in subjects with advanced DR (14·0%, 14·22 x 1000 PYs) as compared to those with non-advanced DR (5·1%, 4·99 x 1000 PYs; overall logrank 31·89; p<0·0001); pairwise over strata logrank statistic provides the following results: non-advanced DR vs. DR-, logrank 4·24 (p=0·039); advanced DR vs. DR-, logrank 31·71 (p<0·0001), and vs. non-advanced DR, logrank 8·86 (p=0·003). Percentages of death and Cox proportional unadjusted HRs (95% CI) are shown vs. DR- (p<0·0001).
